# Screening of Osteogenic-Enhancing Short Peptides from BMPs for Biomimetic Material Applications

**DOI:** 10.3390/ma9090730

**Published:** 2016-08-25

**Authors:** Kei Kanie, Rio Kurimoto, Jing Tian, Katsumi Ebisawa, Yuji Narita, Hiroyuki Honda, Ryuji Kato

**Affiliations:** 1 Department of Basic Medicinal Sciences, Graduate School of Pharmaceutical Sciences, Nagoya University, Furo-cho, Chikusa-ku, Nagoya 464-8601, Aichi, Japan; kanie-k@ps.nagoya-u.ac.jp; 2 Graduate School of Pure and Applied Sciences, University of Tsukuba, 1-1-1 Tennodai, Tsukuba 305-8577, Ibaraki, Japan; KURIMOTO.Rio@nims.go.jp; 3 Biomaterials Unit, International Center for Materials Nanoarchitectonics (WPI-MANA), National Institute for Materials Science (NIMS), 1-1 Namiki, Tsukuba 305-0044, Ibaraki, Japan; 4 Department of Biotechnology, Graduate School of Engineering, Nagoya University, Furo-cho, Chikusa-ku, Nagoya 464-8603, Aichi, Japan; tian.jing@j.mbox.nagoya-u.ac.jp (J.T.); honda@nubio.nagoya-u.ac.jp (H.H.); 5 Department of Plastic and Reconstructive Surgery, Nagoya University Graduate School of Medicine, 65 Turumai-cho, Showa-ku, Nagoya 466-8550, Aichi, Japan; ebisawa@med.nagoya-u.ac.jp; 6 Department of Cardiac Surgery, Nagoya University Graduate School of Medicine, 65 Turumai-cho, Showa-ku, Nagoya 466-8550, Aichi, Japan; ynarita@med.nagoya-u.ac.jp

**Keywords:** osteogenic peptides, peptide array, umbilical cord mesenchymal stem cells, bone morphogenetic proteins, bone regeneration

## Abstract

Bone regeneration is an important issue in many situations, such as bone fracture and surgery. Umbilical cord mesenchymal stem cells (UC-MSCs) are promising cell sources for bone regeneration. Bone morphogenetic proteins and their bioactive peptides are biomolecules known to enhance the osteogenic differentiation of MSCs. However, fibrosis can arise during the development of implantable biomaterials. Therefore, it is important to control cell organization by enhancing osteogenic proliferation and differentiation and inhibiting fibroblast proliferation. Thus, we focused on the screening of such osteogenic-enhancing peptides. In the present study, we developed new peptide array screening platforms to evaluate cell proliferation and alkaline phosphatase activity in osteoblasts, UC-MSCs and fibroblasts. The conditions for the screening platform were first defined using UC-MSCs and an osteogenic differentiation peptide known as W9. Next, in silico screening to define the candidate peptides was carried out to evaluate the homology of 19 bone morphogenetic proteins. Twenty-five candidate 9-mer peptides were selected for screening. Finally, the screening of osteogenic-enhancing (osteogenic cell-selective proliferation and osteogenic differentiation) short peptide was carried out using the peptide array method, and three osteogenic-enhancing peptides were identified, confirming the validity of this screening.

## 1. Introduction

Bone formation and regeneration are necessary phenomena in situations such as fracture due to osteoporosis, cleft lip and palate (CLP) and surgery (for example, cardiac surgery, plastic surgery and cancer surgery). Osteoporosis is characterized by low bone mineral density, strength and micro-architectural deterioration, leading to an increased risk of fragility fractures [1]. With an increasing aging population, the number of osteoporosis fractures is expected to increase in the near future. Fracture risk due to osteoporosis is as high as 40%; these fractures occur in the spine, hip or wrist and often decrease the quality of life. CLP is immediately recognizable by the disruption of normal facial structure [2]. Although not a major cause of mortality in developed countries, CLP causes considerable morbidity in the affected children. CLP causes problems with feeding, speaking and hearing that can be corrected to varying degrees by surgery and dental treatment. The development of bone graft materials for treating CLP has been reported in a clinical study [3]. Many materials similar to bone have been used in surgery. For example, in cardiac surgery, bone wax and sternal pins are used. Bone wax is used as a physical barrier to maintain hemostasis on the surface edges of bones in cardiac operations [4]. Sternal pins are used to reinforce sternal closure and sternal stability [5]. However, there are limitations to each of these materials. Bone wax may induce a foreign body reaction and mechanically inhibit osteoblast activity [4]. Sternal pins made of poly-l-lactide may exhibit a lack of osteoconductivity and the ability to fuse with bone. Although these materials have been widely studied for bone healing [6,7], developing materials that enhance bone regeneration remains a challenge.

Mesenchymal stem cells (MSCs) are one of the most promising resources in regenerative medicine and tissue engineering. MSCs derived from different sources, such as bone marrow (BM), adipose tissue (AT) and umbilical cord (UC), have been extensively studied for bone cell therapy and tissue engineering [8]. Particularly, UC-MSCs exhibit multipotent stem cell characteristics and can differentiate into osteoblasts, chondrocytes, neurons and endothelial cells. One of the advantages of UC-MSCs compared to other sources of MSCs is that they can be obtained by less invasive methods without harming the mother or infant [9] and are generally included in clinical waste that is routinely discarded. Several studies have shown that UC-MSCs have the ability for osteogenic differentiation [10,11], but not adipogenic differentiation [9].

Bone morphogenetic proteins (BMPs) are a family of growth factors known to induce bone formation and have been studied in the context of osteogenic differentiation in MSCs [12]. Since the discovery of BMPs in 1965 [13], the central role of BMPs in human skeletal remodeling has been identified in numerous in vitro, in vivo and clinical studies [14,15]. More than 40 members of the BMP family have been identified, and BMPs have been implicated in the healing of osteoporotic fractures because of their key role in osteogenic differentiation and bone formation. Among the many different BMPs, BMP-2, BMP-4, BMP-6 and BMP-7 are the most studied in osteoporosis and have been associated with its pathways [14]. Many studies have shown that BMP-2, BMP-4, BMP-6 or BMP-7 can regulate osteogenic differentiation of MSCs and bone regeneration both in vitro and in vivo [16,17,18,19,20]. The sequences of BMPs are also highly conserved across species. BMP-2 exhibits knuckle epitopes, and the peptides derived from these epitopes are presently considered as promising replacements for BMPs [21]. For instance, the knuckle epitope of BMP-2, KIPKASSVPTELSAISTLYL, induces elevated alkaline phosphatase (ALP) activity in osteo-progenitor cells and in vivo calcification [22]. Additionally, hydrogel-immobilized peptides (KIPKASSVPTELSAISTLYL peptide and the cell adhesion peptide, RGD) affected osteogenic differentiation and mineralization in progenitor bone marrow stromal cells [23]. Several BMP-7-derived peptides that encourage the mineralization process in osteoblasts have also been identified [24,25] and used for a PLGA polymer that enhances osteogenic differentiation of MSCs [26]. Previous studies comparing BMP-2 and BMP-7 suggested that certain bioactive areas in these proteins are similar in function and amino acid sequence [24,25]. Additionally, a novel peptide from another region of BMP-7 was named as bone forming peptide-1; animals transplanted with bone-forming peptide-1-treated MSCs showed a strong increase in bone formation [27]. Moreover, osteogenic peptides that are not derived from BMP sequences have been identified. A RANKL-binding peptide, W9 (YCWSQYLCY), is known to inhibit osteoclastogenesis and induce osteoblast differentiation and mineralization in pre-osteoblastic cells [28]. A positively-charged 14-amino acid growth peptide (ALKRQGRTLYGFGG) identical to the C-terminus of histone H4 was identified as an osteogenic growth peptide [29]. The osteogenic effect of osteogenic growth peptide has been investigated both in vitro and in vivo (in primary human osteoblast culture [30] and in a rabbit model [31]).

However, fibrosis can arise during the development of implantable biomaterials. Fibrosis is the final and common pathological outcome of many chronic inflammatory diseases. It is defined by excessive accumulation of fibrous connective tissue (components of the extracellular matrix, such as collagen and fibronectin) in and around the inflamed and damaged tissue, resulting in fatal organ damage [32]. For instance, in the intestine, the mechanisms of fibrosis include fibroblast (FB) proliferation and migration and recruitment of FBs differentiated from MSCs by activated growth factors induced by inflammation [33,34]. Therefore, it is important to control FB proliferation, migration and MSC differentiation to prevent the fibrosis caused by biomaterial implantation. MSCs themselves may be useful for treating fibrotic diseases, particularly TNF-stimulated gene 6 protein as a mediator of anti-inflammatory effects [35]. For instance, biomaterial implantation, cell-selective control to inhibit FB proliferation and enhancing the proliferation and osteogenic differentiation of osteogenesis-related cells (MSC and osteoblasts (OBs)) to inhibit differentiation to FB is essential in osteogenesis and bone regeneration. However, few studies have examined biomaterial development and biomolecule screening that include the concept of cell-selectivity.

Thus, we focused on screening of osteogenic-enhancing peptides to enhance osteogenic proliferation and differentiation and inhibit FB proliferation as bone-regenerative biomaterials. For peptide screening, we used the SPOT synthesis peptide array method [36,37]. We developed our original cell-peptide interaction screening, which includes a peptide array-based interaction assay for solid-bound peptides and anchorage-dependent cells (PIASPAC) [38,39]. In our previous study, several cell-selective adhesion peptides were identified, including the endothelial cell (EC)-selective adhesion peptide, and the effect of EC-selectivity has been investigated on a poly(ε-caprolactone) polymer, both in vitro and in vivo [40,41,42].

**Figure 1:**
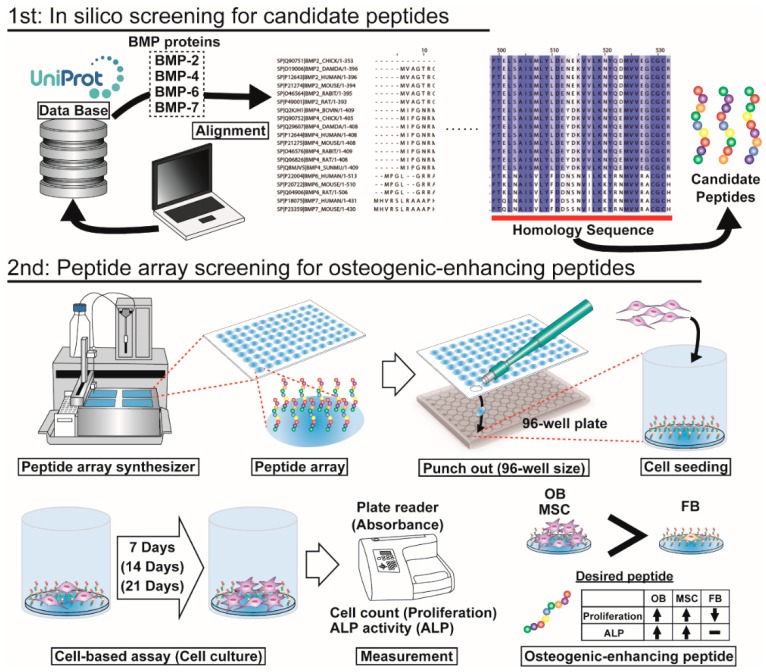
Schematic of this study. First, in silico screening of candidate peptides was performed. From the protein database UniProt, the sequences of several BMPs, such as BMP-2, BMP-4, BMP-6 and BMP-7, from several species were obtained. The protein sequences were aligned in the UniProt database, and homologous sequences were defined. From the homologous sequences, candidate 9-mer peptides were selected for the next peptide array screening. Second step, peptide array screening of osteogenic-enhancing peptides was conducted. A peptide array was fabricated using a peptide array synthesizer and was punched out in the 96-well plate size in a 96-well plate. Cells were seeded and cultured for several days. Cell proliferation and osteogenic differentiation were detected by cell counting and ALP activity, respectively. The desired peptides exhibit higher proliferation of osteoblasts (OBs) and MSCs than of fibroblasts (FBs) and high ALP activity in OBs and MSCs.

In the present study, we developed a new peptide-screening platform to evaluate osteogenic enhancement (osteogenic cell-selective proliferation and osteogenic differentiation) and identified several osteogenic-enhancing short peptides. To develop the new osteogenic peptide screening platform, ALP activity, which is one of the most famous assays for initial osteogenesis, was adopted as the case study. Before screening, the conditions of the screening platform were defined using the W9 peptide, which is an example of the shortest sequence known to be an osteogenic differentiation peptide. Additionally, UC-MSCs were chosen to detect lower ALP activity. Next, in silico screening was first performed to define candidate peptides and to search the homology of several BMPs related to osteogenic differentiation in several species (Figure 1). The length of the candidate peptide was decided to compare the W9 peptide that is a same-length peptide. Candidate 9-mer peptides showing highly homologous sequences to BMPs were screened and selected. Subsequently, screening of osteogenic-enhancing peptide was carried out using the peptide array method (Figure 1). The cell types used in this screening were related to bone regeneration and fibrosis and included UC-MSCs, OBs and FBs. In this study, a screening platform for osteogenic-enhancing peptide was established, and the effect and validity of these peptides were investigated.

## 2. Results

### 2.1. Conditions of Peptide Array Screening

**Figure 2:**
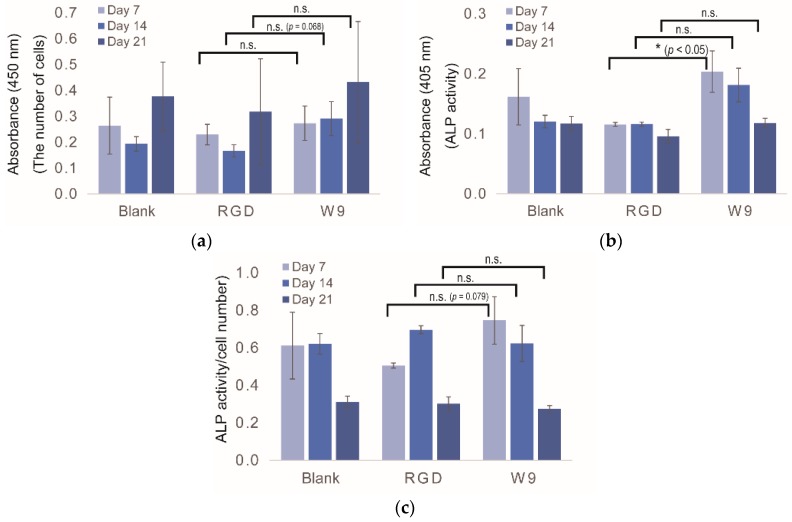
Conditions used for the peptide array screening. UC-MSCs were used for the experiment. (**a**) Proliferation assay by WST-8; (**b**) ALP activity assay; (**c**) ALP activity/cell number. Each assay was performed at Days 7, 14 and 21. Blank indicates no peptide. All experiments were performed in triplicate (* *p* < 0.05, n.s.: *p* ≥ 0.05).

To screen for osteogenic-enhancing peptides using a peptide array, it is necessary to determine the optimal day for evaluation. Thus, cell proliferation and osteogenic differentiation assays with UC-MSCs were carried out at Days 7, 14 and 21 on the peptide array. As controls, no peptide (Blank), RGD peptide and W9 peptide were used. RGD peptide is well known as a cell adhesion peptide, while W9 is known to be an osteogenic differentiation peptide. In the proliferation assay, no differences were observed between Blank and RGD at all time points (Figure 2a). For the W9 peptide, UC-MSCs showed better proliferation than with the RGD peptide, particularly at Day 14 (*p* = 0.068). The ALP assay showed no differences between Blank and RGD at all time points (Figure 2b). However, for the W9 peptide, a significant difference was observed compared to the RGD peptide, particularly at Day 7 (*p* < 0.05). To define the more significant endpoint of the ALP activity assay, the ALP activity per unit cell number was calculated (Figure 2c). The value of W9 at Day 7 was slightly different from that of the RGD peptide at the same time points (*p* = 0.079). Considering that ALP is an osteogenic marker in the initial stage, the duration of the peptide array screening was defined as Day 7.

### 2.2. In Silico Screening to Determine Candidate Peptides

For in silico screening, several sequences of BMPs were downloaded from UniProt. The information regarding the BMPs used in this study is listed in Table 1. Four BMP families (BMP-2, -4, -6 and -7) and eight species (CHICK, DAMDA, HUMAN, MOUSE, RAT, BOVINE, RABBIT and SUNMU) were included, and 19 BMPs were selected in total. BMP-2 protein was from six species; BMP-4 was from eight species; BMP-6 was from three species; and BMP-7 was from two species. Thus, BMP-2 and BMP-4 were the most commonly-investigated proteins for osteogenic differentiation.

**Figure 3:**
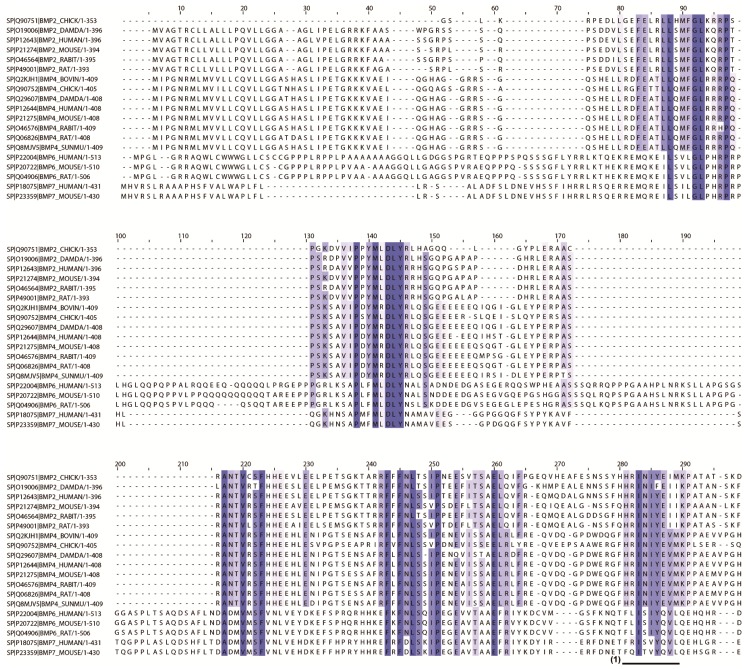

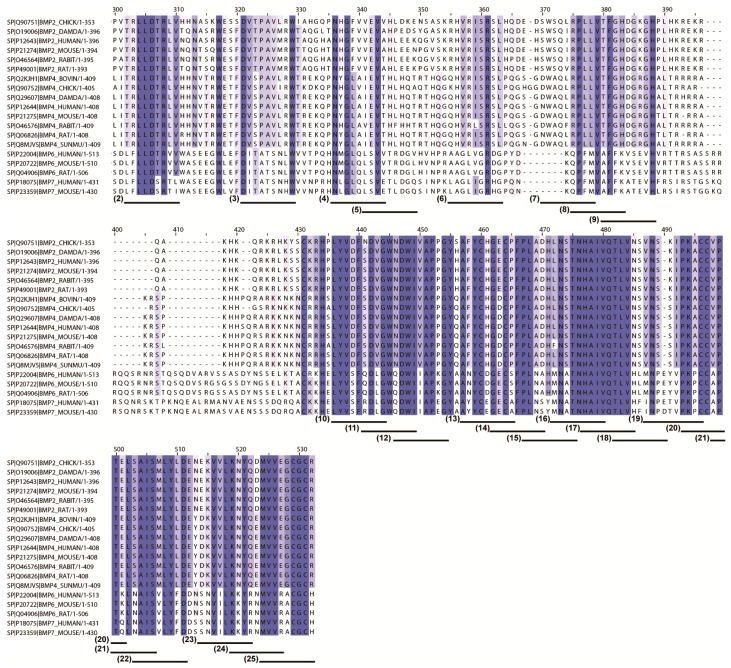
Homology analysis of BMP sequences for screening candidate peptides in silico. Darker blue-colored amino acids indicate high homology, and lighter blue-colored amino acids indicate low homology. Numbered underlines at the bottom of the peptide sequence alignment indicate the 25 peptide sequences selected for peptide array screening (Underlines (1) to (25)). For example, Underline (1) indicates the peptide sequence HRINIYEII (derived from BMP-2), which is listed in Table 2 as the No. 1 peptide.

To determine the candidate peptides for peptide array screening, 19 BMP sequences were aligned to define the important sequences that may be related to osteogenic differentiation. To select candidate peptides, we set two criteria depending on the following hypotheses: (1) homologous BMP sequences across several species with the ability for osteogenic enhancement; and (2) short peptide-like 9-mers with the ability for osteogenic enhancement. Figure 3 shows the alignment and homology analysis results. The homologous sequences were found in the middle or at the end of the C-terminal region of the BMPs. From this analysis, 25 candidate peptides were selected from the homologous regions of nine consecutive amino acids. The 25 candidate peptides are listed in Table 2. The actual peptide sequences are based on human BMP-2.

### 2.3. Peptide Array Screening for Osteogenic Proliferation Peptides

**Figure 4:**
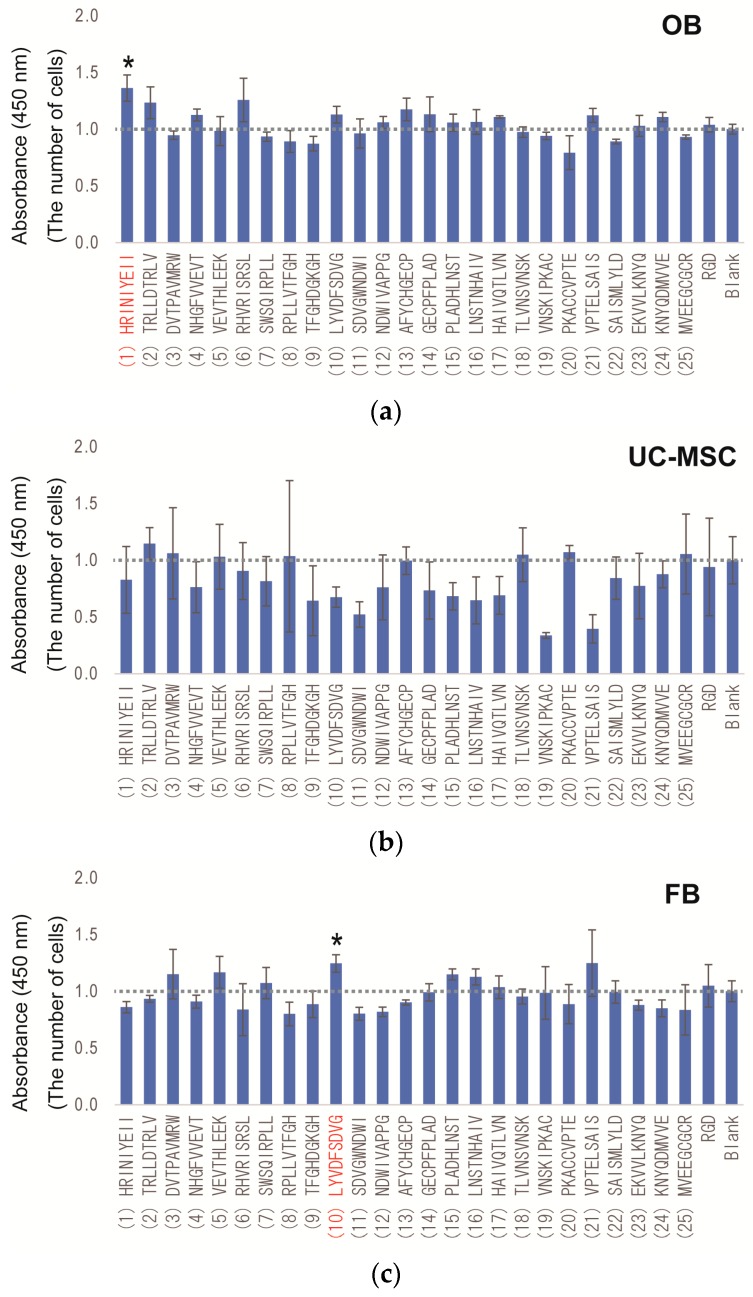
Results of the cell proliferation assay. The number of cells was calculated by the WST-8 assay at Day 7. The value was normalized to Blank (value of no peptide = 1.0). (**a**) OB; (**b**) UC-MSC; and (**c**) FB. All experiments were performed in triplicate. * Denotes statistical significance compared to Blank (no peptide), *p* < 0.05, Student’s *t*-test.

To identify the osteogenic-enhancing peptides in 25 candidate peptides, all peptides, including RGD and the Blank, were synthesized on cellulose membranes by fluorenyl-meth oxy-carbonyl (F-moc) solid phase peptide synthesis, as previously reported [40,41]. In this screening, cell-selective osteogenic proliferation and osteogenic differentiation assays were performed to identify the osteogenic-enhancing peptides. Figure 4 shows the results of the cell proliferation assay in OBs, UC-MSCs and FBs. In OB proliferation peptide screening, several peptides (Nos. 1, 2, 4, 6, 10, 12, 13, 14, 15, 16, 17, 21, 23, 24 and RGD) showed higher proliferation than the no peptide Blank (Figure 4a). Proliferation of peptide No. 1 was significantly higher than that in the Blank (*p* < 0.05). In the screening for UC-MSC proliferation peptides, several peptides (Nos. 2, 3, 5, 8, 18, 20 and 25) showed higher proliferation than the no peptide Blank (Figure 4b). However, none of the peptides were statistically different from the Blank. In the screening for FB proliferation peptides, 17 peptides (except for Nos. 3, 5, 7, 10, 15, 16, 17 and 21) showed lower proliferation than the Blank (Figure 4c). Only peptide No. 10 showed significantly higher proliferation than that of the Blank (*p* < 0.05). In contrast, peptide No. 11 showed significantly lower proliferation than that in the Blank (*p* < 0.05). These results indicate variation in proliferation because the peptides were derived from BMPs in each cell type and that several peptides, such as peptide No. 2 (showing higher proliferation of OBs and UC-MSCs and lower proliferation of FBs, respectively) and Nos. 1, 6 (showing higher proliferation of OBs and lower proliferation of FBs, respectively) could be considered as osteogenic cell-selective proliferation peptides.

### 2.4. Peptide Array Screening for Osteogenic Differentiation Peptides

To identify osteogenic differentiation peptides, an ALP activity assay was performed. Figure S1 shows the results of the ALP assay in OBs and UC-MSCs. In the screening for osteogenic differentiation peptides for OB, several peptides (Nos. 4, 6, 8, 10, 11, 16, 17, 18, 20, 22 and RGD) showed higher differentiation than the Blank (Figure S1a). Differentiation with peptide Nos. 4, 6 and 16 was significantly higher than that in the Blank (*p* < 0.05). In the screening for osteogenic differentiation peptides for UC-MSC, 15 peptides (except for Nos. 2, 7, 8, 11, 13, 14, 18, 20, 21, 23 and 24) showed higher differentiation than in the Blank (Figure S1b); however, none of the peptides showed a statistically-significant difference compared to the Blank.

**Figure 5:**
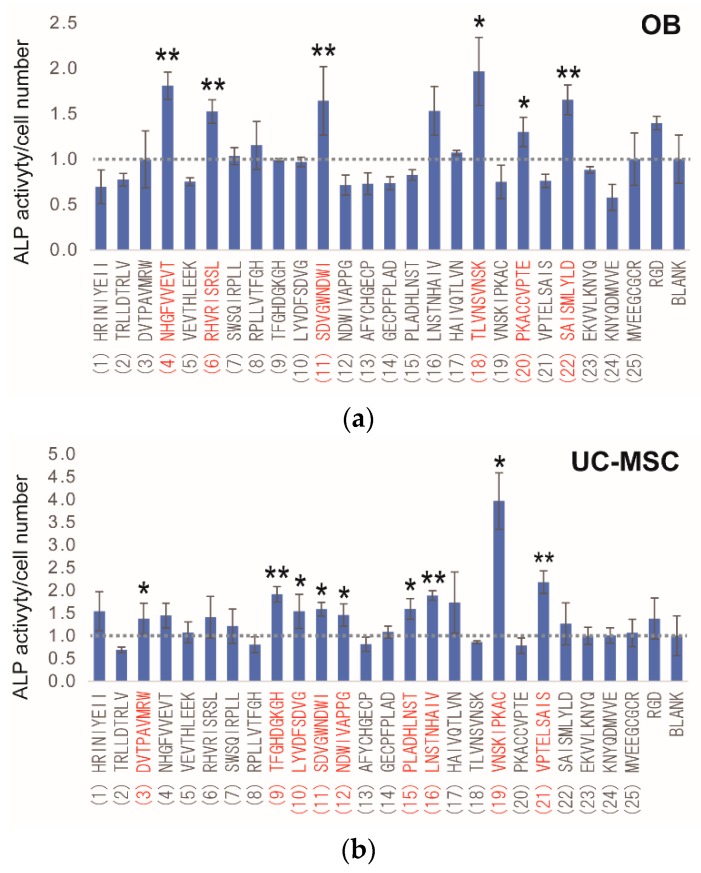
Results of ALP activity per unit cell number. The value was calculated by dividing the ALP activity by cell number and normalized to Blank (value of no peptide = 1.0). (**a**) OBs; (**b**) UC-MSCs. All experiments were performed in triplicate. * Denotes statistical significance compared to Blank (no peptide), *p* < 0.05, Student’s *t*-test. ** Denotes statistical significance compared to Blank, *p* < 0.01, Student’s *t*-test.

**Figure 6:**
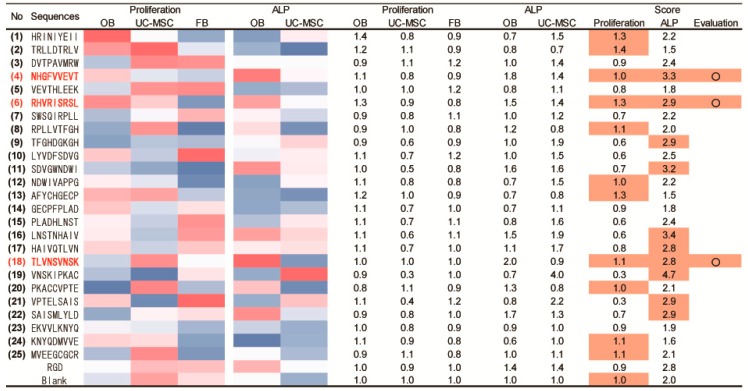
Screening results for osteogenic-enhancing peptides. The heat map indicates the values of cell proliferation and ALP activity. Red denotes a higher value of proliferation or ALP activity, whereas blue indicates a lower value. Score indicates the reference value denoting the osteogenic-enhancing peptides. The value of the proliferation score was calculated to subtract the FB from the sum of the OB and UC-MSC values (ex. No. 1 HRINIYEII peptide: 1.4 + 0.8 − 0.9 = 1.3). The value of the ALP score was calculated as the sum of the OB and UC-MSC values (ex. No. 1 HRINIYEII peptide: 0.7 + 1.5 = 2.2). The orange color indicates a higher value than that of the RGD peptide.

When investigating ALP activity, it is important to consider ALP activity per unit cell number. The number of cells may be higher for the peptide defined as having higher ALP activity. To resolve this problem, all ALP values were divided by unit cell numbers. Figure 6 shows the results of the ALP assay per unit cell number in OBs and UC-MSCs. In OBs, 12 peptides (Nos. 4, 6, 7, 8, 11, 16, 17, 18, 20, 22, 25 and RGD) showed higher differentiation than the Blank (Figure 5a). Differentiation with peptide Nos. 4, 6, 11, 18, 20, 22 and RGD was significantly higher than in the Blank (*p* < 0.05). In UC-MSCs, 20 peptides (except for Nos. 2, 8, 13, 18, 20 and 23) showed higher differentiation than the Blank (Figure 5b), and differentiation with peptide Nos. 2, 9, 10, 11, 12, 15, 16, 19 and 21 was significantly different than in the Blank (*p* < 0.05).

These results indicate that several osteogenic differentiation peptides are derived from BMPs, and several of these peptides may be considered as osteogenic differentiation peptides, such as peptide No. 11 (showing higher ALP activity in both OBs and UC-MSCs).

### 2.5. Determination of Osteogenic-Enhancing Peptides

To identify osteogenic-enhancing peptides (osteogenic cell-selective proliferation (higher proliferation of OBs and UC-MSCs and lower for FBs) and osteogenic differentiation (higher ALP activity in OBs and UC-MSCs)), each peptide was scored based on its osteogenic cell-selective proliferation and differentiation-inducing capacity. Figure 6 shows the results of this determination. The Score colored in orange indicates higher values compared to those of the RGD peptide. In osteogenic cell-selective proliferation, 11 peptides (Nos. 1, 2, 4, 6, 8, 12, 13, 18, 20, 24 and 25) showed a higher score than that of the RGD peptide. In osteogenic differentiation, 10 peptides (Nos. 4, 6, 9, 11, 16, 17, 18, 19, 21 and 22) showed a higher score than that of the RGD peptide. To combine these results, three peptides (No. 4: NHGFVVEVT; No. 6: RHVRISRSL; and No. 18: TLVNSVNSK) were selected as osteogenic-enhancing peptides with both the ability for osteogenic cell-selective proliferation and osteogenic differentiation. These results indicate that osteogenic-enhancing peptides are derived from BMP sequences according to the original peptide array method developed in the present study.

## 3. Discussion

In the present study, we developed a new PIASPAC method to screen for osteogenic-enhancing peptides. Upon aligning 19 BMPs (BMP-2, BMP-4, BMP-6 and BMP-7) from eight species, 25 candidate 9-mer peptides were obtained from in silico analysis. Using the established PIASPAC method, three osteogenic-enhancing peptides were discovered in the osteogenic cell-selective proliferation assay and osteogenic differentiation assay.

As shown in Figure 2b, ALP activity with the W9 peptide was approximately 1.3- to 2.0-fold higher than that of the no peptide Blank and RGD peptide. Similar results were observed in a previous study wherein the value of ALP activity with the W9 peptide was found to be 1.3- to 3.0-fold higher than that with the no peptide Blank [28]. Although the conditions used in the previous study differed from those used in our study (the type of MSCs, surface-immobilized peptide, etc.), our new PIASPAC method using a peptide array was similar to that used in the previous study regarding ALP activity.

**Figure 7:**
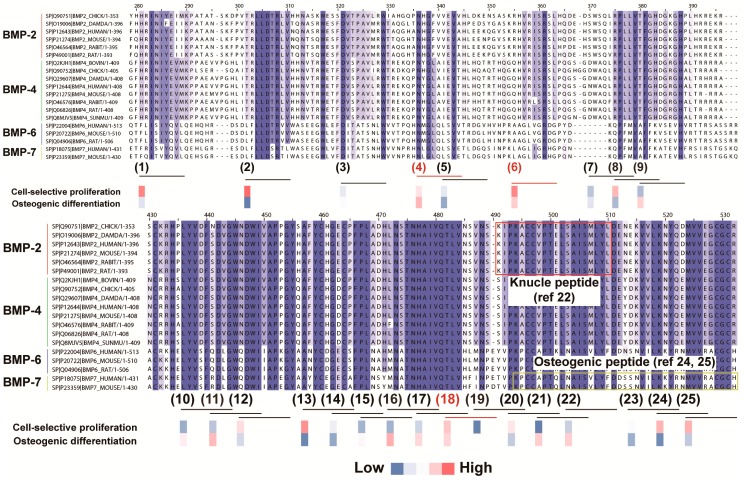
Location of osteogenic-enhancing peptides. The results of osteogenic cell-selective proliferation and osteogenic differentiation peptides and their locations in the BMP sequences are indicated.

To select screening candidates, we hypothesized that osteogenic-related sequences (peptides) existed in regions showing high homology across the 19 types of BMPs from eight species. Several homologous regions were observed in the in silico screening process (Figure 3). Furthermore, these regions were present in the end region of the C-terminal in BMP sequences. In a previous study, osteogenic-related sequences (peptides) were detected in end region sequences, such as in the knuckle peptide from BMP-2 and osteogenic peptides from BMP-7 (Figure 7) [22,24,25].

As a result of peptide screening, three peptides were found to be osteogenic-enhancing peptides from among the 25 candidate peptides (Figure 5). To identify the location of the functional peptides including these three peptides, we investigated the location of these functional peptides in the protein sequence and determined the functional area that could induce osteogenic cell-selective proliferation and osteogenic differentiation. Figure 7 shows the location of the candidate peptides and their functions (osteogenic cell-selective proliferation and osteogenic differentiation). As described above, highly functional peptides, such as peptide Nos. 18 and 19, were found to be located near the knuckle peptide or osteogenic peptide as reported previously [22,24,25]. Moreover, peptide Nos. 21 and 22, which were not selected as osteogenic cell-selective peptides, showed a higher ability for osteogenic differentiation. These four peptides (Nos. 18, 19, 21 and 22) are near or overlapping the knuckle peptide and osteogenic peptide. Additionally, novel peptides that are not related to the knuckle peptides were identified (peptide Nos. 4 and 6) as having an osteogenic-enhancing function. These results suggest that our original method using the peptide array is useful for screening osteogenic-enhancing peptides.

In this study, our results supported two hypotheses: (1) homologous sequences of BMPs across several species are involved in osteogenic-enhancing; and (2) short peptides, such as 9-mers, are involved in osteogenic enhancement. As a result, three short peptides with the ability of osteogenic enhancement were identified from homologous BMP sequences. Although these results supported our hypotheses, further studies are needed to evaluate mature osteogenesis in vitro and in vivo using alizarin red staining, calcium determination, hydroxyapatite determination and mRNA expression analysis related to the bone regeneration gene (such as RUNX2, osteocalcin and collagen I). However, human cells were used in the present study, so it is necessary to investigate the same experiment with other species’ cells, such as mouse or rat. Additionally, it is necessary to investigate other peptides other than the 25 candidate peptides, and the efficiency should be evaluated to compare using sequences of BMPs and that of another protein family. Moreover, it is necessary to investigate the inhibition of fibrosis in vivo to confirm the effect of osteogenic cell-selective proliferation. However, the concept of cell-selectivity is a novel and important idea for tissue engineering for controlling regeneration and its side effects, such as fibrosis. The peptide array platform developed in this study is a promising method for identifying cell-selective peptides and aiding the development of biomimetic materials.

## 4. Materials and Methods

### 4.1. In Silico Screening for Candidate Peptides

The BMP sequences were obtained from UniProt and were then aligned in the database. The graphical image was obtained using Jalview (ver. 2.9.0b2), which is a free program for multiple sequence alignment editing.

### 4.2. Cell Culture

Normal human osteoblasts (CC-2538, LONZA, Basel, Switzerland) were maintained in the OGM Bullet Kit (CC-3207, LONZA). Human mesenchymal stem cells from umbilical cord matrix (C-12971, Promo Cell, Heidelberg, Germany) were maintained in mesenchymal stem cell growth medium (C-28010, Promo Cell). Normal human dermal fibroblasts (KF-4109, KURABO, Osaka, Japan) were maintained in Dulbecco’s modified Eagle’s medium (DMEM) (044-29765, Wako Pure Chemical Industries, Osaka, Japan) containing 10% fetal bovine serum (Nichirei Biosciences, Tokyo, Japan) and 1% penicillin-streptomycin (168-23191, Wako Pure Chemical Industries). The cells were maintained at 37 °C with 5% CO_2_ and were used within 4 to 6 passages.

### 4.3. Peptide Array Synthesis

A peptide array was designed and synthesized using F-moc synthesis following the manufacture’s protocol using a peptide synthesizer (ASP222, Intavis AG, Köln, Germany) with some modifications, as reported previously [40,41]. All F-moc amino acids, F-moc 11-amino undecanoic acid (linker) and other organic solvents were purchased from Watanabe Chemical Industries (Hiroshima, Japan). In the array for the cellular assay, peptide sequences were synthesized in triplicate for each array.

### 4.4. Cell Proliferation Assay Using the Peptide Array

The previously-described PIASPAC protocol was applied to assay the relative cell number of OBs, UC-MSCs and FBs with some modifications [40,41]. Viable cells were seeded on the cell assay platform as droplets (OB, FB: 1.4 × 10^4^ cells/cm^2^, UC-MSC: 2.9 × 10^4^ cells/cm^2^). After 7 days of culture, WST-8 (Water soluble Tetrazolium salts) (CK04, DOJINDO, Kumamoto, Japan) was added to the medium following the manufacture’s protocol for 1 h, and the absorbance of the supernatant was measured using an absorption plate reader (iMark™ Microplate Absorbance Reader, 1681130JA, Bio-Rad, Hercules, CA, USA) at 450 nm. Significant differences in the data between two conditions were determined by the Student’s *t*-test.

### 4.5. ALP Activity Assay Using the Peptide Array

The previously-described PIASPAC protocol was applied to assay the relative ALP activity in OBs and UC-MSCs with some modifications [40,41]. Viable cells were seeded on the cell assay platform as droplets (OB: 1.4 × 10^4^ cells/cm^2^, UC-MSC: 2.9 × 10^4^ cells/cm^2^). After 7 days of culture, SIGMAFAST™ *p*-nitrophenyl phosphate tablets (N1891, SIGMA-ALDRICH, St. Louis, MO, USA) dissolved in water were added to the medium following the manufacturer’s protocol and incubated for 30 min. The absorbance of the supernatant was measured by an absorption plate reader (iMark™ Microplate Absorbance Reader) at 405 nm. Significant differences in the data between two conditions were determined by Student’s *t*-test.

## 5. Conclusions

In this study, a novel peptide-screening platform using a peptide array was developed to identify osteogenic-enhancing peptides. For this screening, in silico analysis was performed to compare the sequences of 19 types of BMPs that are well known as bone regenerative molecules. As a result, 25 candidate 9-mer peptides were selected from homology sequences, including some known to be from an osteogenic differentiation region. Among the 25 short peptides, three peptides were found to be osteogenic-enhancing peptides through peptide array-based osteogenic cell-selective proliferation and osteogenic differentiation assays.

## Figures and Tables

**Table 1:** BMPs used in alignment analysis.

No.	Entry	Entry Name	Protein Names	Organism	Length
1	Q90751	BMP2_CHICK	Bone morphogenetic protein 2 (BMP-2) (fragment)	*Gallus gallus* (chicken)	353
2	O19006	BMP2_DAMDA	Bone morphogenetic protein 2 (BMP-2)	*Dama dama* (fallow deer) (*Cervus dama*)	396
3	P12643	BMP2_HUMAN	Bone morphogenetic protein 2 (BMP-2) (bone morphogenetic protein 2A) (BMP-2A)	*Homo sapiens* (human)	396
4	P21274	BMP2_MOUSE	Bone morphogenetic protein 2 (BMP-2) (bone morphogenetic protein 2A) (BMP-2A)	*Mus musculus* (mouse)	394
5	O46564	BMP2_RABIT	Bone morphogenetic protein 2 (BMP-2)	*Oryctolagus cuniculus* (rabbit)	395
6	P49001	BMP2_RAT	Bone morphogenetic protein 2 (BMP-2) (bone morphogenetic protein 2A) (BMP-2A)	*Rattus norvegicus* (rat)	393
7	Q2KJH1	BMP4_BOVIN	Bone morphogenetic protein 4 (BMP-4)	*Bos taurus* (bovine)	409
8	Q90752	BMP4_CHICK	Bone morphogenetic protein 4 (BMP-4)	*Gallus gallus* (chicken)	405
9	Q29607	BMP4_DAMDA	Bone morphogenetic protein 4 (BMP-4)	*Dama dama* (fallow deer) (*Cervus dama*)	408
10	P12644	BMP4_HUMAN	Bone morphogenetic protein 4 (BMP-4) (bone morphogenetic protein 2B) (BMP-2B)	*Homo sapiens* (human)	408
11	P21275	BMP4_MOUSE	Bone morphogenetic protein 4 (BMP-4) (bone morphogenetic protein 2B) (BMP-2B)	*Mus musculus* (mouse)	408
12	O46576	BMP4_RABIT	Bone morphogenetic protein 4 (BMP-4)	*Oryctolagus cuniculus* (rabbit)	409
13	Q06826	BMP4_RAT	Bone morphogenetic protein 4 (BMP-4) (bone morphogenetic protein 2B) (BMP-2B)	*Rattus norvegicus* (rat)	408
14	Q8MJV5	BMP4_SUNMU	Bone morphogenetic protein 4 (BMP-4) (sBmp4)	*Suncus murinus* (Asian house shrew) (musk shrew)	409
15	P22004	BMP6_HUMAN	Bone morphogenetic protein 6 (BMP-6) (VG-1-related protein) (VG-1-R) (VGR-1)	*Homo sapiens* (human)	513
16	P20722	BMP6_MOUSE	Bone morphogenetic protein 6 (BMP-6) (VG-1-related protein) (VGR-1)	*Mus musculus* (mouse)	510
17	Q04906	BMP6_RAT	Bone morphogenetic protein 6 (BMP-6) (VG-1-related protein) (VGR-1)	*Rattus norvegicus* (rat)	506
18	P18075	BMP7_HUMAN	Bone morphogenetic protein 7 (BMP-7) (osteogenic protein 1) (OP-1) (eptotermin alfa)	*Homo sapiens* (human)	431
19	P23359	BMP7_MOUSE	Bone morphogenetic protein 7 (BMP-7) (osteogenic protein 1) (OP-1)	*Mus musculus* (mouse)	430

**Table 2:** Candidate peptides for the peptide array screening.

No.	Peptide Sequences
(1)	HRINIYEII
(2)	TRLLDTRLV
(3)	DVTPAVMRW
(4)	NHGFVVEVT
(5)	VEVTHLEEK
(6)	RHVRISRSL
(7)	SWSQIRPLL
(8)	RPLLVTFGH
(9)	TFGHDGKGH
(10)	LYVDFSDVG
(11)	SDVGWNDWI
(12)	NDWIVAPPG
(13)	AFYCHGECP
(14)	GECPFPLAD
(15)	PLADHLNST
(16)	LNSTNHAIV
(17)	HAIVQTLVN
(18)	TLVNSVNSK
(19)	VNSKIPKAC
(20)	PKACCVPTE
(21)	VPTELSAIS
(22)	SAISMLYLD
(23)	EKVVLKNYQ
(24)	KNYQDMVVE
(25)	MVEEGCGCR
	RGD
	Blank (no peptide)

## Supplementary Materials

The following are available online at www.mdpi.com/1996-1944/9/9/730/s1. Figure S1: Results of the cell activity assay. The osteogenic differentiation of cells was determined by the ALP assay at Day 7. The values were normalized to that of the Blank (value of no peptide = 1.0). (a) OBs; (b) UC-MSCs. All experiments were performed in triplicate. * Denotes statistical significance compared to Blank (no peptide), *p* < 0.05, Student’s *t*-test. ** Denotes statistical significance compared to Blank, *p* < 0.01, Student’s *t*-test.
