# Supplementary material for: Screening of Osteogenic-Enhancing Short Peptides from BMPs for Biomimetic Material Applications
Source: Materials (Basel). 2016 Aug 25;9(9):730. doi: 10.3390/ma9090730 (PMC5457080; doi:10.3390/ma9090730)
Supplement: Supplementary file 1 [file materials-09-00730-s001.pdf]

Materials **2016**, *9*, 730; doi:10.3390/ma9090730

Supplementary Materials: Screening of Osteogenic Enhancing Short Peptides from BMPs for Biomimetic Material Applications

Kei Kanie, Rio Kurimoto, Jing Tian, Katsumi Ebisawa, Yuji Narita, Hiroyuki Honda and Ryuji Kato

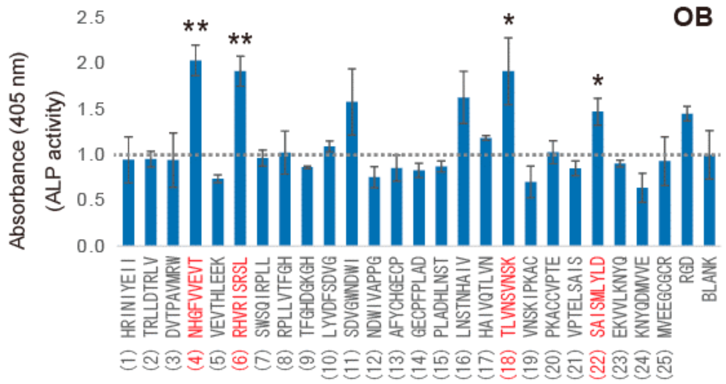

(**a**)

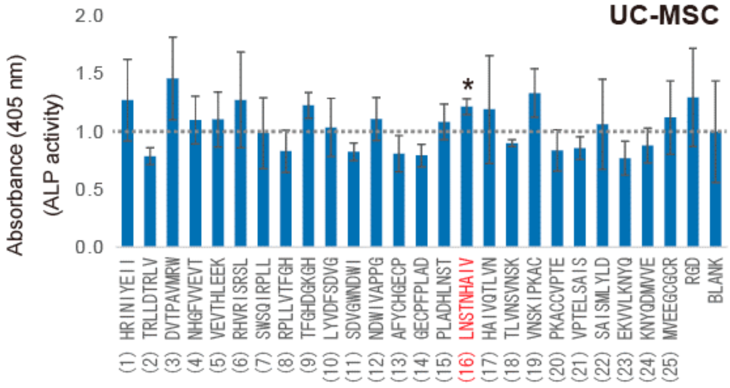

(**b**)

Figure S1. Results of the cell activity assay. The osteogenic differentiation of cells was determined by the ALP assay at Day 7. The values were normalized to that of the BLANK (value of no peptide = 1.0). (**a**) OBs; (**b**) UC-MSCs. All experiments were performed in triplicate. * Denotes statistical significance compared to BLANK (no peptide), $p < 0.05$, Student's *t*-test. ** Denotes statistical significance compared to BLANK, $p < 0.01$, Student's *t*-test.
